# Adult T-cell leukemia/lymphoma in the Caribbean cohort is a distinct clinical entity with dismal response to conventional chemotherapy

**DOI:** 10.18632/oncotarget.10223

**Published:** 2016-06-22

**Authors:** Monica Zell, Amer Assal, Olga Derman, Noah Kornblum, Ramakrishna Battini, Yanhua Wang, Deepa M. Narasimhulu, Ioannis Mantzaris, Aditi Shastri, Amit Verma, Hilda Ye, Ira Braunschweig, Murali Janakiram

**Affiliations:** ^1^ Albert Einstein College of Medicine, Bronx, NY, USA; ^2^ Department of Oncology, Montefiore Medical Center/ Albert Einstein College of Medicine, Bronx, NY, USA; ^3^ Department of Pathology, Montefiore Medical Center/ Albert Einstein College of Medicine, Bronx, NY, USA; ^4^ Department of Cell Biology, Albert Einstein College of Medicine, Bronx, NY, USA

**Keywords:** human T-cell lymphotropic virus type-1 (HTLV-1), adult T-cell leukemia/lymphoma (ATLL), non-Hodgkin lymphoma, T-cell lymphoma, allogeneic stem cell transplantation

## Abstract

Adult T-cell leukemia/lymphoma (ATLL) is a rare and aggressive disease caused by human T-cell lymphotropic virus type 1 that predominantly affects Japanese and Caribbean populations. Most studies have focused on Japanese cohorts. We conducted a retrospective analysis of 53 cases of ATLL who presented to our institution between 2003-2014. ATLL in the Caribbean population presents more often as the acute and lymphomatous subtypes, is associated with complex cytogenetics, and has a high rate of CNS involvement. The overall response rate to first-line therapies with anthracycline-based regimens was poor (32%), with a median survival of only 6.9 months. A complete or partial response to first-line regimens was associated with better survival. There was no difference in survival between patients who received chemotherapy alone versus chemotherapy with antiviral agents. Allogeneic transplantation was performed in five patients, two of whom achieved complete remission despite residual or refractory disease. Recipients of allogeneic transplantation had significantly improved overall survival compared to non-transplanted patients. This is the first analysis to describe ATLL pathological features, cytogenetics, and response to standard therapy and transplantation in the Caribbean cohort.

## INTRODUCTION

Adult T-cell leukemia/lymphoma (ATLL) is a rare mature T-cell malignancy first described in Japan in 1977 [1]. The causative agent of ATLL has been identified as the human T cell lymphotropic virus type 1 (HTLV-1) [2–4], which is endemic to Japan, the Caribbean islands, Central and South America, and parts of sub-Saharan Africa [5]. Prevalence in the United States reflects emigration patterns of individuals from endemic areas. ATLL develops in approximately 5% of HTLV-1 infected individuals who acquire the virus via vertical transmission [5].

The pathogenesis of ATLL in HTLV-1-infected individuals may differ from one population to another. In a comparison between Japanese and Caribbean cohorts, Japanese patients displayed a higher incidence of ATLL, male predominance, and older peak age of onset [6]. Another study demonstrated differences in ATLL disease development, where antibody titers and anti-tax antibody, both markers of strong immune response and viral replication and the latter, an antibody to a viral protein involved in ATLL pathogenesis [7], were higher in Jamaican subjects [6]. In contrast, a high viral load with low antibody titers was found in Japanese subjects, which is a pattern consistent with high risk for ATLL. Clinically, ATLL is divided into four distinct subtypes based on the Shimoyama diagnostic criteria: acute, lymphomatous, chronic, and smoldering [8]. The acute and lymphomatous subtypes have poorer prognosis and survival [8]. Major prognostic factors associated with a shorter survival in all types of ATLL include elevated lactate dehydrogenase (LDH) level, advanced age, greater than three involved lesions, hypercalcemia, poor performance status, serum albumin < 3.5 g/dL, and sIL-2R > 20,000 U/mL [9–11]. Although there is no specific karyotypic or molecular abnormality in ATLL, many common cytogenetic abnormalities have been described in Japanese studies [12–14]. There have been no large series analyzing cytogenetic findings in Caribbean patients.

Survival of ATLL patients remains poor despite therapy. The regimen with the best outcomes as reported in a randomized trial by the Japanese Clinical Oncology Group (JCOG) is VCAP-AMP-VECP (vincristine, cyclophosphamide, doxorubicin, and prednisone (VCAP), doxorubicin, ranimustine, and prednisone (AMP), and vindesine, etoposide, carboplatin, and prednisone (VECP)), also known as LSG15 [15]. When compared to CHOP administered biweekly, a higher response rate was noted (40% versus 25%, p=0.02), as well as a trend for higher three-year survival (24% versus 13%, p=0.085). The use of this regimen has been limited in the United States, and the National Comprehensive Cancer Network (NCCN) guidelines recommend regimens typically used in other non-Hodgkin lymphomas (NHL) such as CHOP, CHOEP, and dose-adjusted EPOCH [16]. While still not a standard of care, allogeneic hematopoietic stem cell transplantation can be considered [17–19], while the use of autologous stem cell transplantation is not recommended [17, 20].

Given the limited scope of published data on Caribbean ATLL, a current and complete description is needed to further characterize the nature of this entity as distinct from the Japanese variant. We report the clinicopathologic features, treatment patterns, and disease outcomes of ATLL in a large, predominantly Caribbean cohort from a single institution in Bronx, New York.

## RESULTS

### Demographic and baseline data

We identified 53 cases of ATLL treated at Montefiore Medical Center between 2003 – 2014 [Table 1]. Median age at presentation was 54 years (range 28-87), with a female predominance (62%). Our cohort almost entirely represents “western” ATLL, with 91% of patients of Caribbean or Latin origin (n=48); the remaining five patients were of African origin. No patients were of non-Hispanic Caucasian ethnicity or Asian descent.

**Table 1:** Patient demographics and baseline clinical features

Characteristic	Total frequency (%)
Age at diagnosis (yrs)	
40 or less	11 (21%)
41 – 60	24 (45%)
61 or greater	18 (34%)
Gender	
Female	33 (62%)
Male	20 (38%)
Geographic Origin	
Caribbean/Hispanic	48 (91%)
African	5 (9%)
ATLL Subtype	
Acute	36 (68%)
Lymphomatous	14 (26%)
Chronic	3 (6%)
Smoldering	0 (0.0%)
Symptoms and pathological features	
Lymphadenopathy	41 (77%)
Rash	16 (30%)
Pleural Effusion	21 (40%)
Ascites	11 (21%)
Bone Marrow Involvement (40)^a^	31 (78%)
Bone Lesions (51)^a^	7 (14%)
CNS Involvement (27)^a^	9 (33%)
Hypercalcemia	31 (59%)
Lymphocytosis	27 (51%)
Abnormal cytogenetics (22)^a^	13 (59%)
Immunophenotype	
Typical (CD4+/CD8-)	44 (83%)
Atypical (CD4+/CD8+ or CD4-/CD8-)	9 (17%)

a Total number of patients tested for this variable in parentheses. (n=53).

Based on the Shimoyama classification, 36 (68%) patients had acute subtype, 14 (26%) had lymphomatous, and three (6%) had chronic/smoldering disease. Patients presented with generalized lymphadenopathy (77%), bone marrow involvement (58%), hepatosplenomegaly (57%), skin (30%), lung (21%), gastro-intestinal (17%), or bone lesions (14%). Of the 27 patients whose CSF was tested for CNS involvement at presentation, nine were positive (33%). Patients with acute and chronic subtypes tended to present with lymphocytosis (51%) with a mean value of 17,490 (600 – 193,000) cells/microliter. Elevated LDH was seen in all patients with a mean value of 1299 (252 – 9610) units/L. Hypercalcemia was also a common feature (64%), with a mean value of 12.03 (7.4 – 20) mg/dL. Of the patients tested for HIV (70%), none were positive. The majority of patients had the classic CD4+/CD8- CD25+ ATLL immunophenotype (83%), while fewer had an atypical phenotype (CD4+/CD8+ or CD4-/CD8-) (17%).

Of 53 total patients, 15 (28%) had positive CSF cytology and/or flow cytometry at any time during their disease. Three had clinical CNS symptoms (20%), and five had evidence of CNS disease on radiographic imaging (33%) [Table 2]; all patients with CNS symptoms had positive radiographic findings.

**Table 2:** Features of patients with CNS disease

Patient	CNS Symptoms	CSF Flow cytometry / Cytology	Imaging at time of CNS disease
1	None	Paucicellular cytospin specimen with rare atypical lymphocytes	CT head - Negative
2	None	CD4+, CD25+, CD7-, CD26-	CT head - Negative
3	Cranial nerve palsy	CD3 +, CD4+, CD25+, CD27+, CD7-, CD26-	MRI Brain - Diffuse dural thickening
4	None	CD2+, CD3+, CD4+, CD5+, CD7-	CT head - Negative
5	None	23% CD4+ T-cells with reduced CD7 expression, partial expression of CD25	CT head-Negative
6	None	Positive for malignant cells,	MRI head-Meningeal enhancement
7	None	Abnormal CD4+ T-cells that lack CD7	CT Head - Negative
8	Headache	Positive for malignant cells	CT head-Right frontal skull lesion with dehiscence of the inner table, with a minimal epidural component
9	None	CD2+, CD3+, CD4+, CD5+, CD8+, CD7-	MRI Brain - Normal
10	None	CD4+, CD25+, CD7- phenotype	MRI Brain - Faint thin dural enhancement
11	None	CD4+, CD25+ T-cells	CT head - Negative
12	Headache	0.6% CD3+, CD4+, CD7-, CD25+ T-cells	MRI Brain - Large destructive sinonasal mass, extension to adjacent structures. Parameningeal extension.
13	None	CD2+, CD7+, CD3+, CD5+, CD4-, CD8-	CT head - Negative
14	None	9% T-cells CD4+, CD7-, mostly CD25+	CT head - Negative
15	None	Atypical. Scattered lymphocytes, some with mild atypia.	CT head - Negative

CNS: central nervous system; CSF: cerebral spinal fluid; CT: computed tomography; MRI: magnetic resonance imaging.

(n=15).

Out of 22 patients tested for cytogenetic abberations, 10 had complex karyotypes, and three patients had abnormalities limited to one chromosome [Table 3]. Aneuploidy of several chromosomes was noted including +3, −4, −6, −7, +7, +8, −9, −10, −11, −12, −13, −14, +14, −15, −16, −17, −19, +19, −20, +20, +21, −22, +22, +X, and –Y. Deletions affecting chromosomes 1, 3, 5, 6, 7, 8, 9, 11, 12, and 20 were noted. The most commonly affected chromosome was 14, with abnormalities found in eight patients, followed by chromosomes 1, 9, 11, and 20, with abnormalities found in six patients each. Recurrent deletions noted were deletion 3q21, 5q33, and 20q11.2. Recurrent loci affected by additions or duplications included 2p11.2 and 14q32. Recurrent loci affected by translocation or addition included 1q21, 7q36 (one translocation and one addition), 9p13 (one translocation and one addition), 11q13 (one translocation and one duplication), 14q10 (one translocation and one inversion), 19p13.3 (one translocation and one addition), and 20q11.2 (one translocation, one addition and one deletion). Seven patients also had additional unidentified chromosomes.

**Table 3:** Cytogenetic findings in tested patients with ATLL

Subtype	Age/ Gender	Source	Karyotype
Acute	39 / F	BM	48~49, XX, +X, del(1)(p34), add(2)(p11.2), del(3)(q21), dic(3:3)(q21;p21), del(5)(q13q33), del(7)(p13), +8, del(9)(p13), dup(11)(q13q23), −12, −15, −16, −17, add(19)(p13.3), −20, +22
Acute	42 / M	BM	44, X, -Y, −6, der(13)t(13;14)(q10;q10), +mar
Acute	69 / M	BM	48~49, XY, +X, t(1;7)(q32;q36), add(9)(p24), t(11;19)(q13;p13.3), add(14)(p10), +14, add(20)(q11.2), del(20)(q11.2), +20
Acute	35 / M	PB	45~48, X, -Y, der(1)t(1;10)(q21;p11.2), t(1;11)(q22;q24), +der(1)del(1)(q21q42), +3, −4, del(6)(q13q22), add(7)(q36), del(8)(q22q24.3), −10, add(12)(q24.3), +del(12)(q13q14), −14, −15, −16, −17, −19, −20, +21, +21, +22, +22, +2mar
Acute	51 / M	BM	46, XY, del(11)(q23)
Acute	44 / M	BM	46, Y, t(X;9) (p22.1;p13), t(1;20) (p22;q11.2), t(2;15) (q33;p12)
Acute	60 / F	BM	46, XX, 14q11.2 and 14q32 polysomy on FISH^a^
Acute	36 / M	BM	47-48, XY, der(2)t(1;2) (q21;q35), add(3)(p21), del (3) (q21), +3, del (6) (q23), −9, −9, −10, add (14) (q32), +1-3mar
Acute	43 / M	PB	48, XY, del(6)(p22), +7, +14, −17, +3mar
Acute	58 / F	BM	47, XX, t(1;13)(p36.3;q33), dup(2)(p11.2p14), t(4:9)(q34;q31.1), t(15;19)(p11.2;q13.2), +19
Acute	54 / F	BM	84~85, XX, add(3)(q12)x2, +3, add(4)(q31), −4, del(5)(q15q33)x2, −7, add(9)(p13)x2, −10, −10, −11, add(11)(p15), −12, −13, −13, −13, add(13)(p10), −14, i(14)(q10), −15, add(15)(p10), −19, −19, −20, add(20)(q13.3)x2, i(21)(q10)x2, +10mar
Acute	56 / F	BM	45-46, X, add(X)(q22), t(11;15) (p11.2;q12), del(12) (p12), add(14) (p11.2), −16, −17, −22, +mar
Lymphomatous	62 / F	BM	46, XX, del(20)(q11,.2)

a Fluorescent in situ hybridization, this patient had a history of Philadelphia chromosome positive chronic myelogenous leukemia. BM: bone marrow; PB: peripheral blood. (n=13).

### Therapy and survival

Outcomes with front-line therapy for patients with acute or lymphomatous subtype were poor [Table 4]. Most patients (n=42, 79%) received first-line treatment, whereas 17% (n=9) were not treated due to poor performance status, and two patients were lost to follow up after diagnosis. The median age of patients who received treatment was 46 years, while those who were not treated due to comorbidities had a median age of 75 years. One patient with chronic subtype disease received first-line treatment with interferon alpha + zidovudine (IFN + AZT) and achieved complete remission. Among patients with acute or lymphomatous subtype (n=41), chemotherapy alone was given to 23 patients (56%) with an overall response rate (ORR) of 30%. Regimens included EPOCH (n=11), CHOP (n=7), Hyper CVAD (n=4), and CVP (n=1). Chemotherapy with antiviral therapy was administered to 16 patients (39%) with an ORR of 38%. The most common chemotherapy regimen in this group was EPOCH (n=14), while the most common antiviral agents used were lamivudine/zidovudine (n=8), followed by raltegravir (n=5), zidovudine (n=2), and lamivudine (n=1). Two patients were treated with IFN + AZT (5%) alone, with one patient maintaining stable disease over three months, and one with progressive disease. The ORR after first-line therapy for patients with acute or lymphomatous subtype was 32%; nine patients (22%) achieved a CR, four patients (10%) achieved a PR, and eight patients (20%) died. Median duration of response was 171 days after first-line therapy. Of the remaining patients with primary refractory, progressive disease, or relapse (n=39), 56% (n=22) went on to receive second-line therapy, where only two patients (9%) achieved a response. The most common second-line therapy regimens were hyper CVAD (n=5) and ICE (n=4).

**Table 4:** Front-line therapy and response rates in patients with acute or lymphomatous subtypes

Therapy	Number	Overall Response Rate (%)
Chemotherapy Only	23	6 CR, 1 PR (30.4%)
EPOCH	11	4 CR
CHOP	7	2 CR, 1 PR
CVP	1	0
Hyper CVAD	4	0
Chemotherapy with antiviral	16	3 CR, 3 PR (37.5%)
EPOCH + Bortezomib + Raltegravir	5	1 CR, 2 PR
EPOCH + Lamivudine	1	0
EPOCH + Lamivudine / AZT	8	2 CR, 1 PR
CHOP + AZT	1	0
Hyper CVAD + AZT	1	0
Other	2	0 CR, 0 PR (0%)
IFN + AZT	2	0

EPOCH: etoposide, prednisone, vincristine, cyclophosphamide, doxorubicin; CHOP: cyclophosphamide, doxorubicin, vincristine, prednisone; CVP: cyclophosphamide, vincristine, prednisone; Hyper CVAD: hyperfractionated cyclophosphamide, vincristine, doxorubicin, dexamethasone; IFN: interferon alpha; AZT: zidovudine; CR: complete response; PR: partial response.

(n=41).

### Stem cell transplantation

A total of seven patients were treated with hematopoietic stem cell transplantation in our cohort. Autologous transplantation was performed in two chemotherapy-sensitive patients, one with acute subtype and one with lymphomatous, as a consolidation therapy after first-line anthracycline-based chemotherapy (EPOCH and CHOP, respectively). Both patients received BEAM conditioning (carmustine, etoposide, cytarabine, and melphalan). The progression free survival (PFS) after autologous transplant was 13 months and nine months, respectively, with overall survival of 19.8 and 18.5 months, respectively.

Allogeneic hematopoietic stem cell transplantation (allo-HSCT) was performed in five patients [Table 5]. ATLL subtypes of transplanted patients included four acute and one lymphomatous. At the time of transplantation, one patient was in CR, three were in PR, and one had primary refractory disease. Three patients had a matched related donor, one patient a matched unrelated donor, and one a haploidentical donor. After allo-HSCT, two patients achieved a CR and remain in remission at the time of this report, while three patients died. Of those three patients, all died within 100 days of their transplant, with two due to progressive disease and one due to Grade 4 graft versus host disease (GVHD) after immunosuppression withdrawal for disease recurrence.

**Table 5:** Outcomes of allogeneic hematopoietic stem cell transplantation

Patient	1	2	3	4	5
Subtype	Acute	Acute	Acute	Acute	Lymphomatous
Lines of therapy	3	1	1	5	3
Disease status pre-transplant	PR	PR	CR	PD	PR
Graft	Haplo	MRD	MUD	MRD	MRD
Conditioning regimen	RIC	MA	RIC	RIC	RIC
Acute GVHD (Grade)	Skin (2)	GI (4)	GI, Skin (1)	GI (1)	GI (4)
Chronic GVHD	None	N/A	Skin	N/A	N/A
PFS	14.4+	1.4	17.2+	3.7	3.4
OS	25.7+	7.6	28.4+	10.4	47.2

PFS is counted from day of stem cell infusion. OS is counted from index date. PR: partial response; CR: complete response; PD: progressive disease; Haplo: haploidentical donor; MRD: matched related donor; MUD: matched unrelated donor; RIC: reduced intensity conditioning; MA: myeloablative; GVHD: graft versus host disease; PFS: progression free survival; OS: overall survival.

### Overall survival

**Figure 1:**
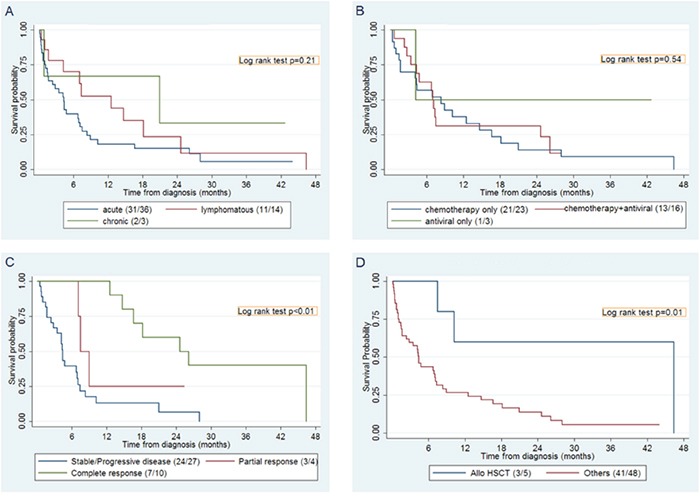
Survival in the ATLL Caribbean cohort **A.** Overall survival by ATLL subtype **B.** Overall survival comparing first-line chemotherapy with antiviral agent versus first-line chemotherapy alone **C.** Overall survival response to first-line treatment **D.** Overall survival of patients with allogeneic hematopoietic stem transplant compared to overall cohort. HSCT: hematopoietic stem cell transplant; allo HSCT: allogeneic hematopoietic stem cell transplant.

The median overall survival (OS) was 6.9 months [Figure 1]. There was no significant difference in survival between patients who received chemotherapy versus chemotherapy with an antiviral agent (p=0.94) [Figure 1b]. Since EPOCH is the most commonly used regimen for ATLL in the United States, we compared survival after therapy with EPOCH-based regimens versus all other regimens and found no statistically significant difference in OS (p=0.21). The response to first-line therapy impacted survival, with those achieving partial (PR) or complete response (CR) experiencing better OS (p<0.01) [Figure 1c]. Compared to those who did not receive an allo-HSCT, a significant increase in OS was observed in allo-HSCT recipients (p=0.02) [Figure 1d].

## DISCUSSION

In this study we outline a tertiary medical center's experience with ATLL, a rare, poor-risk disease with a high mortality rate. Montefiore Medical Center (MMC) in the Bronx serves a large urban population, with many immigrants and descendants of immigrants from the Caribbean. The significant volume of ATLL cases encountered at MMC makes our institution one of the primary centers to diagnose and treat ATLL in the United States. Since most of these patients are from the Caribbean Basin, our experience closely reflects the ATLL experience from that part of the world. This study reveals diagnostic and therapeutic challenges as well as actuarial efficacy of currently accepted treatment strategies. Our study is unique as it describes the natural history, cytogenetics, and outcomes of front-line therapies and stem cell transplantation in a Caribbean ATLL cohort.

ATLL patients diagnosed in the Bronx have a higher incidence of acute and lymphomatous subtypes. The median age at diagnosis is 54 years and approximately 30% of patients have severe concurrent comorbidities/infections or are elderly, making them ineligible for intensive chemotherapy. Interestingly, no patients were co-infected with HIV; whether this is a true association is currently unknown. In this cohort there was a high prevalence of features of higher risk disease (hypercalcemia, high LDH, and bone marrow involvement) [19]. The rate of CNS involvement in this population was as high as 33%, supporting the argument for primary CNS prophylaxis at the time of diagnosis. Despite higher rates of CNS dissemination compared to previously reported incidence in other mature T-cell lymphomas (4.5%) [21], mortality is primarily due to uncontrolled systemic disease. Flow cytometry showed a CD4+/CD8-, CD25+ CD7- T-cell population in most patients, reflecting the typical ATLL immunophenotype. In those with cytogenetic data available, all but three patients had complex karyotypes. Chromosome 14 was the most common chromosome involved, unlike in Japanese cohorts where deletions of chromosome 1 are more common [12]. These complex cytogenetics, markers of cumulative genetic events, and the presence of multiple cell subclones at the time of disease manifestation, likely explain why this disease is highly resistant to standard chemotherapy regimens. Moreover, the tumor suppressor p53 without mutations could be silenced by the pro-viral genome [22] and early and late ATLL could be driven by oncogene versus oncogenic microRNA addiction [23].

First-line chemotherapy for ATLL in the United States is most commonly an anthracycline-based regimen such as CHOP or EPOCH [24]. Multi-agent non-cross-resistant chemotherapy regimens like LSG15 are not used due to non-availability of drugs like ranimustine and vindesine in the United States [25]. Our data suggest that the response to first-line regimens for ATLL is poor, with 68% of patients having progressive or primary refractory disease. Moreover, the achieved responses to these regimens are short-lived and a second remission is difficult to induce. These figures are lower than those reported by Phillips et al [26], but are consistent with historical data and the poor OS (median 6.9 months) of this population. Even though a prior meta-analysis shows benefit with antiviral agents [27], we did not observe any statistically significant difference between patients treated with chemotherapy alone versus chemotherapy and antiviral agents. Only three patients in this cohort were treated with IFN + AZT, and one patient with chronic subtype ATLL achieved a CR. However, the use of antivirals was a favorable factor in those who underwent allogeneic transplantation, and hence we continue to use antiviral therapy in the treatment of all ATLL, especially in the acute subtype.

Seven patients underwent stem cell transplantation. Of the five patients that underwent allo-HSCT, two had a long term CR, still in remission at the time of this report. Unique features in both of these patients included: partially chemotherapy-responsive disease, treatment with antivirals as a part of the induction regimen, and maintenance on IFN+AZT prior to allo-HSCT. Whether these features have an impact on outcomes with allo-HSCT in this population needs to be further investigated. Of the three patients who failed allo-HSCT, two had early relapses within 100 days. The disease responded to withdrawal of immunosuppression in one patient, suggesting a graft-versus-lymphoma effect, though the patient ultimately succumbed to GVHD. A recent non-Japanese ATLL study found improved outcomes in patients who underwent allo-HSCT in comparison to autologous transplant [17], and early application of HSCT may be beneficial [28]. The timing and patient selection for allo-HSCT remain controversial, as its utility could be limited in patients with progressive disease [29]. Based on our experience demonstrating a poor overall survival in these patients, and evidence of a graft vs. lymphoma effect, we propose allo-HSCT in an upfront setting. This would ideally be achieved after first-line treatment with at least a partial response. The conditioning regimen would then provide a window of opportunity for graft vs. lymphoma effects to be established. Autologous transplantation is considered to be ineffective in ATLL. Two patients in our cohort underwent autologous transplantation, with PFS of 13 months and nine months, which were higher than expected for this population. Hence, autologous transplantation may be an option in patients who have achieved at least a partial remission with initial chemotherapy and have evidence of chemosensitive disease. Whether this is related to patient selection or achieving a PR from initial therapy is not known. The OS of 6.9 months in our cohort is less than that of 8.3 and 10.6 months for acute and lymphomatous ATLL, respectively, reported in a larger Japanese cohort [30]. However, in the aforementioned study, 25% of patients who underwent HSCT had long term survival consistent with that of our cohort. The barriers to allogeneic transplantation in this population are 1) lack of matched related donors due to ethnicity, 2) progressive disease and poor response to first-line treatment, and 3) poor performance status due to recurrent infections. Since this was a retrospective analysis the true incidence of other subtypes of ATLL may not be reflected in this study. Moreover, this analysis is limited to a single institution and our experience with ATLL may differ from that of other institutions.

Currently, most studies impacting ATLL management involve Japanese patients. Our study provides insight into the problems faced by the ATLL population in the United States and should focus clinical trials to areas of need accordingly. Urgent clinical trials with improved front-line chemotherapy regimens, in combination with biological agents, are needed to achieve a better quality of response. Differences in HTLV viral load, TAX expression and TCR repertoire of Allo transplant patients in CR vs <CR may be suitable biomarkers to explore in future studies. Allogeneic stem cell transplant should be considered upfront. The timing of antivirals and continuing antivirals/interferon pre- and post-transplant needs to be evaluated. Additionally, T-cell adaptive immunotherapy should be explored in this disease.

## MATERIALS AND METHODS

Patient records were queried using the Clinical Looking Glass ® (CLG) software to identify all cases of HTLV positivity and ATLL by searching pathology and laboratory reports of patients who presented to Montefiore Medical Center (MMC) between 2003 and 2014. CLG is an interactive software application developed at MMC that integrates demographic, clinical, and administrative datasets and allows them to be reproduced in a programmable format for statistical access. Electronic medical records were manually reviewed to confirm diagnoses and for relevant data points. The diagnosis of ATLL was confirmed based on clinical history, pathological findings, and HTLV-1 antibody positivity. Cases were classified by subtype using the Shimoyama criteria [8]. Index date was defined as the date on which a diagnosis of ATLL was made. Demographic data as well as laboratory values, pathologic records, treatment records, outcomes, and survival were collected. Mortality data included death records within our institution as well as those reported by Social Security records. For patients who were discharged to hospice with confirmed documentation of refractory disease and unknown date of death (n=5), date of discharge to palliative care was equated with date of death. All chart review was conducted by one of the study authors and discrepancies were reviewed by at least two authors. This research was approved by our institutional review board and ethics committee. All authors had access to the primary clinical data and were involved in data analysis.

### Statistical analysis

Data generated by CLG and complemented with individual chart review were transferred to a computer spreadsheet (Microsoft Excel, Microsoft Corp., Redmond, WA). For the analysis of categorical variables, we reported proportions and p values calculated with Pearson chi square or Fisher exact test as appropriate. Kaplan-Meier curves were used to compare survival and statistical significance was examined using the log rank test. Statistical analyses were performed with Stata v12 (StataCorp LP, College Station, TX) and a two-tailed alpha of 0.05 was used to denote significance.
